# Supplementary material for: Sustainable Processing of Floral Bio-Residues of Saffron (Crocus sativus L.) for Valuable Biorefinery Products
Source: Plants (Basel). 2021 Mar 11;10(3):523. doi: 10.3390/plants10030523 (PMC8001949; doi:10.3390/plants10030523)
Supplement: Supplementary file 1 [file plants-10-00523-s001.pdf]

plants

MDPI

Article

Sustainable processing of floral bio-residues of Saffron (*Crocus sativus* L.) for valuable biorefinery products

Stefania Stelluti [1], Matteo Caser [1,*], Sonia Demasi [1] and Valentina Scariot [1]

[1] Department of Agricultural, Forest and Food Sciences, University of Torino. Largo Paolo Braccini 2,10095, Grugliasco (TO), Italy; stefania.stelluti@unito.it; matteo.caser@unito.it; sonia.demasi@unito.it; valentina.scariot@unito.it

* Correspondence: matteo.caser@unito.it; Tel.: +39-011-670-8935

Supplementary Table S1. Total phenolic content (TPC), total anthocyanin content (TAC), and antioxidant activity measured with the FRAP, ABTS, and DPPH assays, in dried tepal extracts obtained through maceration (M) and ultrasound assisted extraction (UAE) techniques, and the solvents water or methanol at three concentrations (20% - Met20, 50% - Met50, and 80% - Met80). Statistical comparisons between solvents for both extraction techniques separately are provided.

Extraction		TPC (mgGAE 100g^{-1} DW)	TAC (mgG3G 100g^{-1} DW)	FRAP (mmolFe^{2+} Kg^{-1} DW)	ABTS (µmolTE g^{-1} DW)	DPPH (µmolTE g^{-1} DW)
**M**	**Water**	1142.27 ± 43.52	345.04 ± 132.47	571.54 ± 3.21 a	13.82 ± 0.72	15.56 ± 2.29 b
**M**	**Met20**	1123.53 ± 59.86	268.13 ± 26.76	506.73 ± 13.85 b	14.20 ± 0.60	17.83 ± 2.46 b
**M**	**Met50**	1106.45 ± 9.17	300.39 ± 15.02	535.83 ± 10.30 ab	14.62 ± 0.29	24.52 ± 2.55 a
**M**	**Met80**	1166.96 ± 33.15	249.13 ± 11.97	511.72 ± 22.49 b	14.29 ± 0.32	24.17 ± 1.53 a
p		**ns**	**ns**	**0.03367 ***	**ns**	**0.03446 ***
**UAE**	**Water**	1150.63 ± 11.23	413.30 ± 137.16 a	556.90 ± 11.91 a	12.76 ± 0.81	23.55 ± 3.60
**UAE**	**Met20**	1113.27 ± 46.11	178.39 ± 34.03 c	460.05 ± 35.55 b	13.39 ± 1.46	19.35 ± 4.83
**UAE**	**Met50**	1066.89 ± 26.36	277.09 ± 49.06 ab	506.68 ± 21.80 b	15.10 ± 0.38	24.58 ± 1.46
**UAE**	**Met80**	1153.49 ± 22.74	231.70 ± 30.19 bc	513.67 ± 21.12 ab	14.34 ± 0.81	21.03 ± 1.81
p		**ns**	**0.03446 ***	**0.0329 ***	**ns**	**ns**

Values of mean and standard deviation are reported for each variable. Statistical comparisons were performed using the non-parametric Kruskal-Wallis test. Values with the same letter are not statistically different at $p < 0.05$, according to Dunn's post-hoc test; *, $p < 0.05$; **, $p < 0.01$; ***, $p < 0.001$; ns = not significant.
